# Supplementary material for: Optimizing structured surfaces for diffractive waveguides
Source: Nat Commun. 2025 Jun 6;16:5256. doi: 10.1038/s41467-025-60626-3 (PMC12144198; doi:10.1038/s41467-025-60626-3)
Supplement: Supplementary file 1 — Supplementary Information [file 41467_2025_60626_MOESM1_ESM.pdf]

Supplementary Information

Optimizing Structured Surfaces for Diffractive Waveguides

Yuntian Wang[1,2,3†], Yuhang Li[1,2,3†],Tianyi Gan[1,3], Kun Liao[1,2,3], Mona Jarrahi[1,3] and Aydogan Ozcan[1,2,3*]

[1]Electrical and Computer Engineering Department, University of California, Los Angeles, CA, 90095, USA

[2]Bioengineering Department, University of California, Los Angeles, CA, 90095, USA

[3]California NanoSystems Institute (CNSI), University of California, Los Angeles, CA, 90095, USA

[†]These authors contributed equally to the work

[*]Correspondence to: ozcan@ucla.edu

This PDF file includes:

Supplementary Figures S1-S16

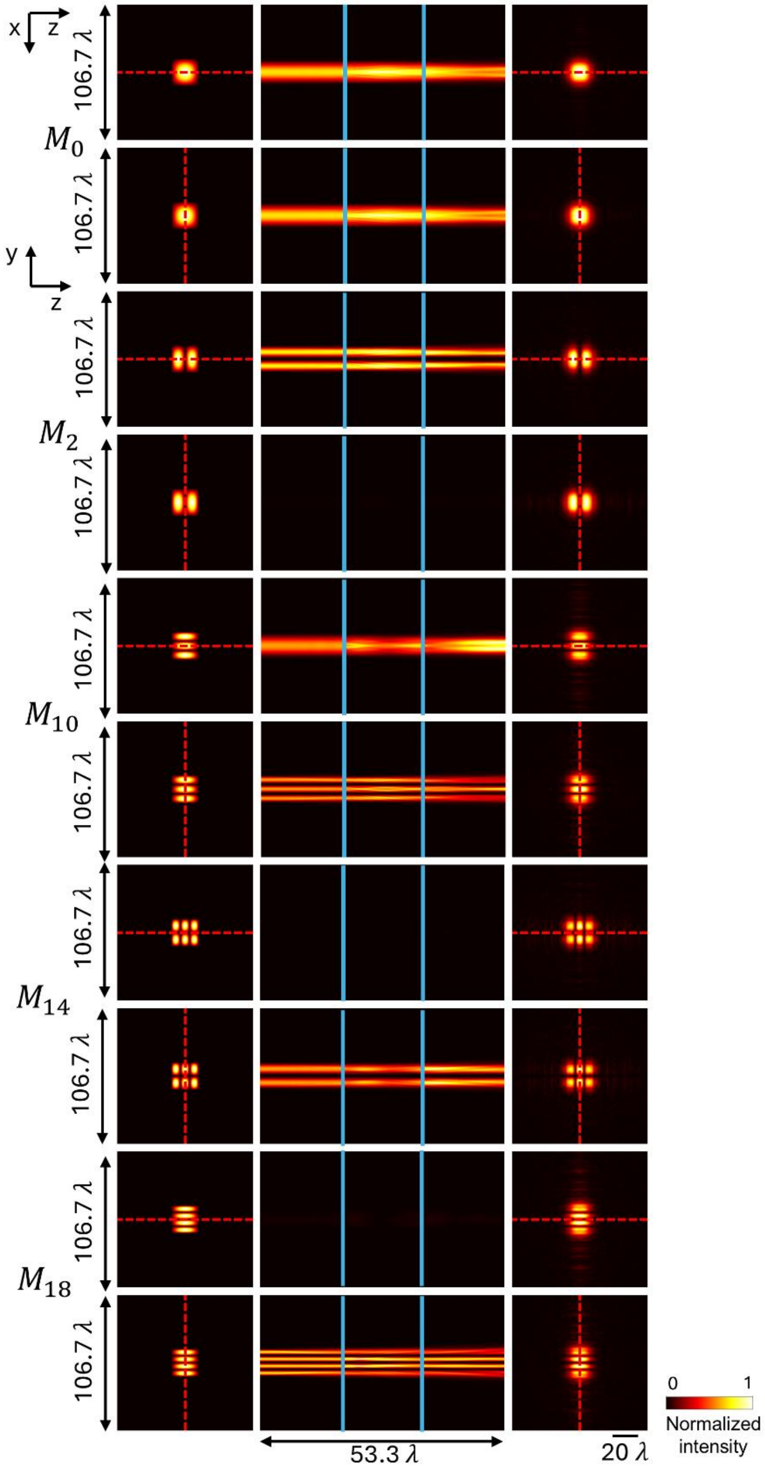

Supplementary Fig. S1. Cross sectional profile of mode propagation within the diffractive waveguide shown in Fig. 1d of main text.

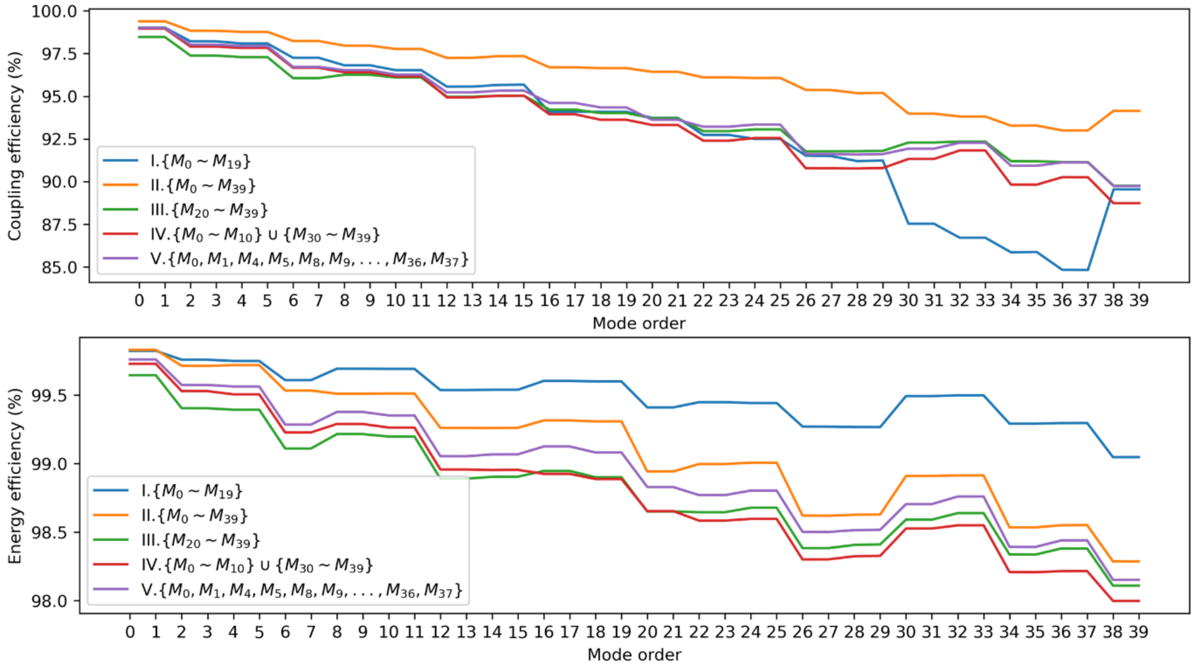

Supplementary Fig. S2. Testing results of diffractive waveguide models trained with different sets of spatial modes. Five sets were used for training: I. 20 lower order modes (M_0~M_{19}, same as in Fig. 2); II. all 40 modes (M_0~M_{39}); III. 20 higher order modes (M_{20}~M_{39}); IV. a combination of 10 lower order modes M_0~M_9 and 10 higher order modes M_{30}~M_{39}; V. two consecutive modes followed by skipping two, i.e., $\{M_0, M_1, M_4, M_5, M_8, M_9, \ldots M_{32}, M_{33}, M_{36}, M_{37}\}$. All the trained diffractive waveguide models were tested with the full set of 40 spatial modes.

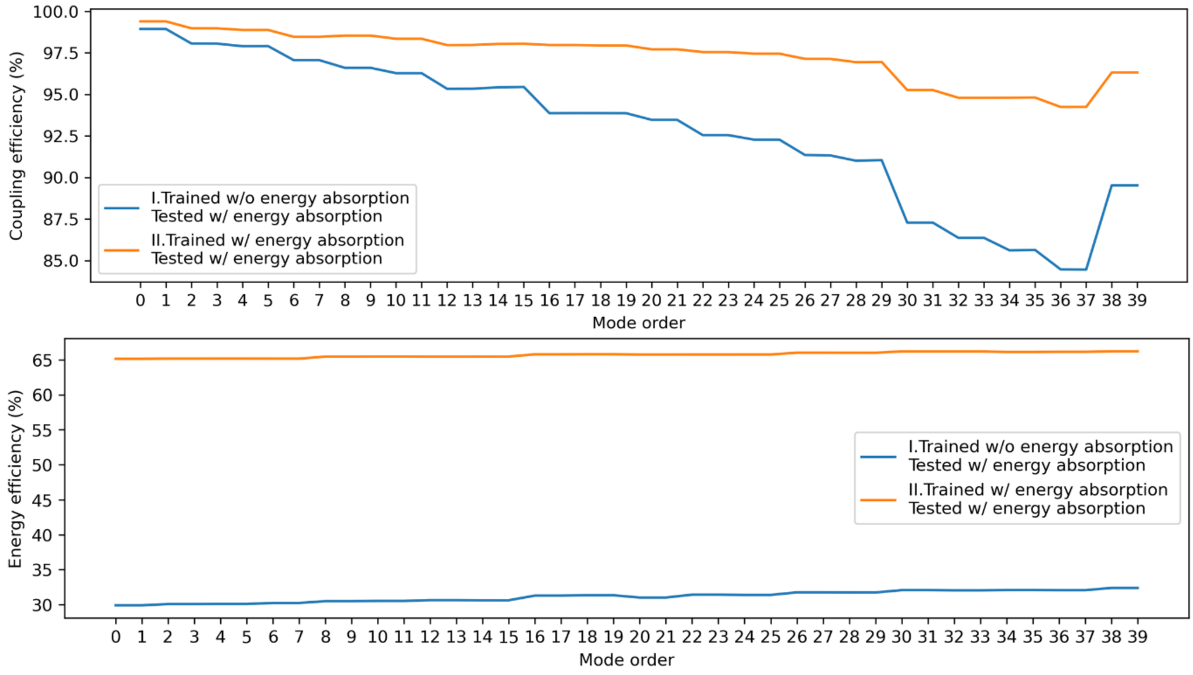

Supplementary Fig. S3. Testing results of the diffractive waveguide models trained with and without incorporating material absorption into the training process. Both diffractive waveguide models were tested with an absorbing material; we assumed λ = 0.75 mm and the complex refractive index of the diffractive layers was assumed to be 1.72 + *j* 0.03, which corresponds to a commonly used 3D printing material (VeroBlack, Objet30 Pro, Stratasys). Lower loss materials can be used to further improve the energy efficiency of these diffractive waveguide designs.

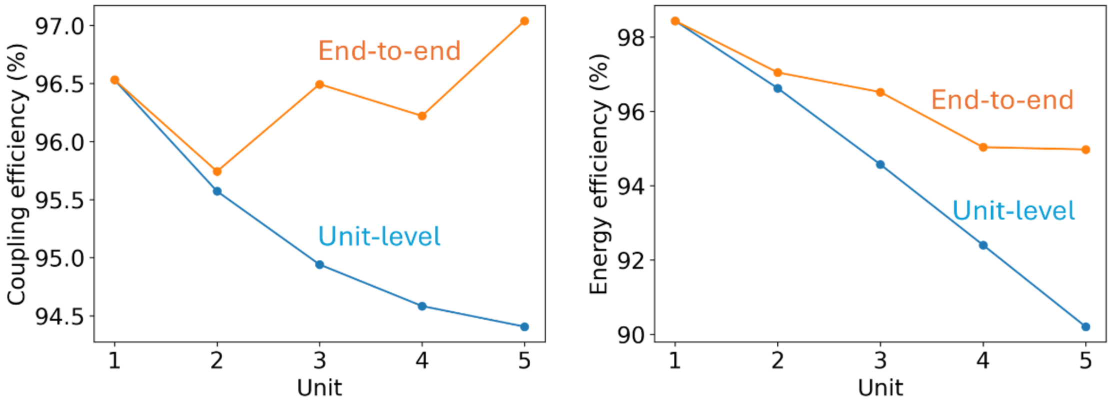

Supplementary Fig. S4. Comparison of the testing performances of unit-level and end-to-end optimization strategies. End-to-end strategy outperforms unit-level design strategy.

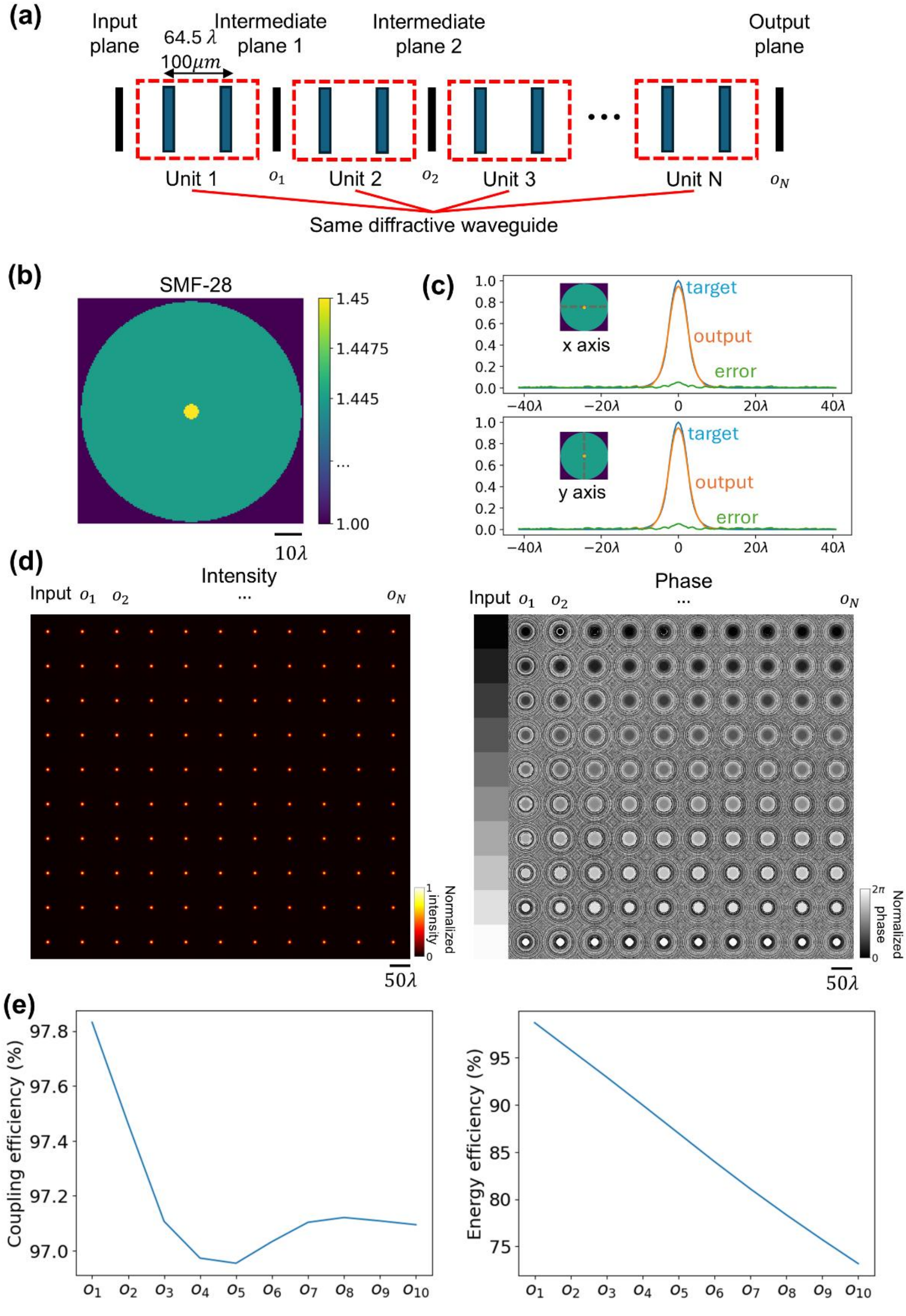

Supplementary Fig. S5. Testing results of a cascadable single-mode diffractive waveguide operating at $\mathbf{1550}\ \boldsymbol{nm}$. (a) Schematic of a cascaded diffractive waveguide for infrared mode transmission at $1550\ nm$ with N identical units. (b) Refractive index profile of the standard SMF-28 waveguide. (c) Output optical

field profile of the dielectric waveguide (target), diffractive waveguide (output) and the error in between. (d) Intensity and phase profiles of the optical fields at the input, intermediate and output planes. (e) Coupling efficiency and energy efficiency of the transmitted optical modes at the intermediate planes and the output plane.

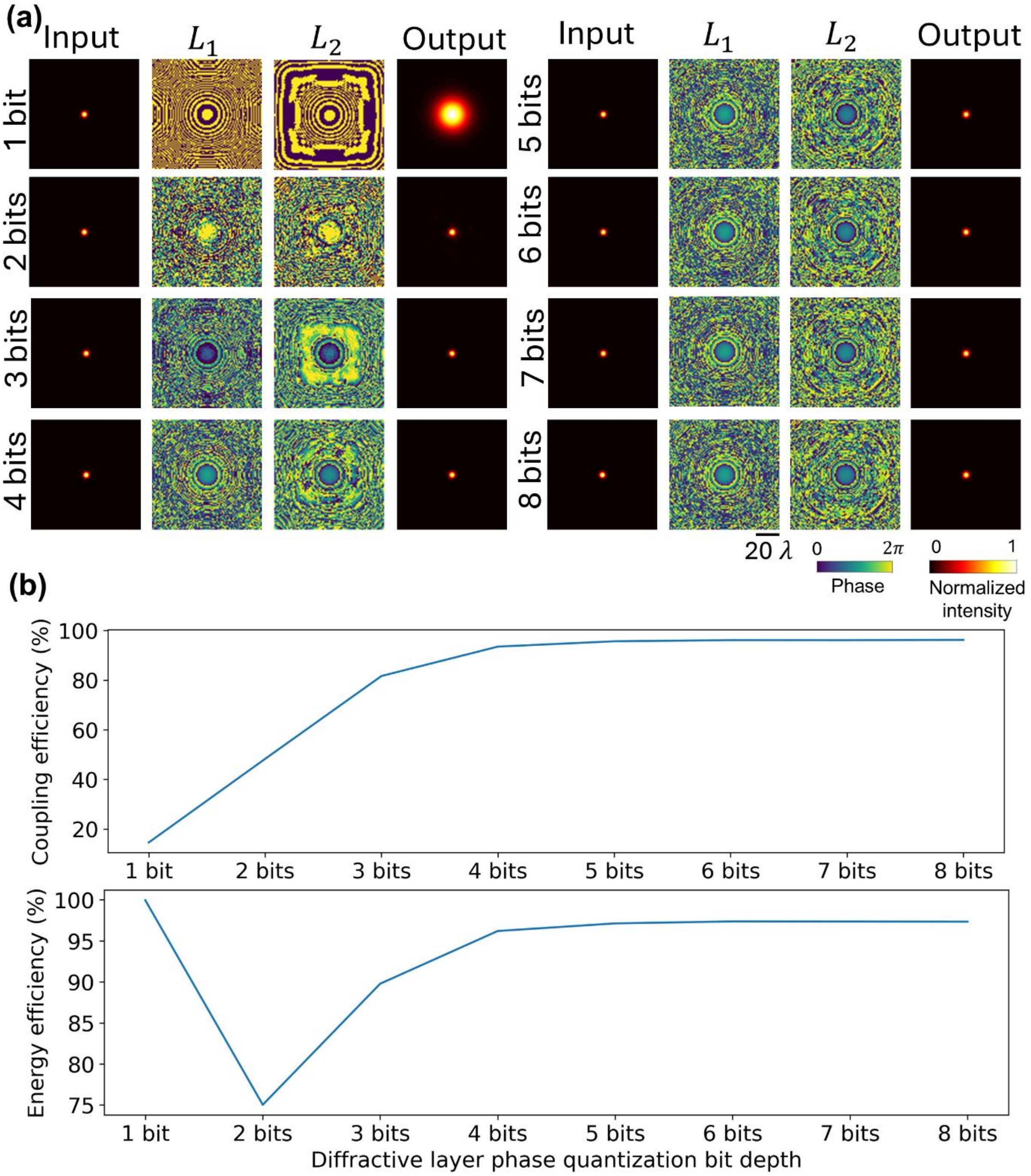

Supplementary Fig. S6. Testing results of a single-mode diffractive waveguide operating at 1550 nm, designed with varying levels of phase quantization bit depths. (a) Input field, output field and diffractive layer phase profiles corresponding to different phase quantization bit depths. (b) Coupling and energy efficiency values of diffractive waveguides designed with different phase quantization bit depths.

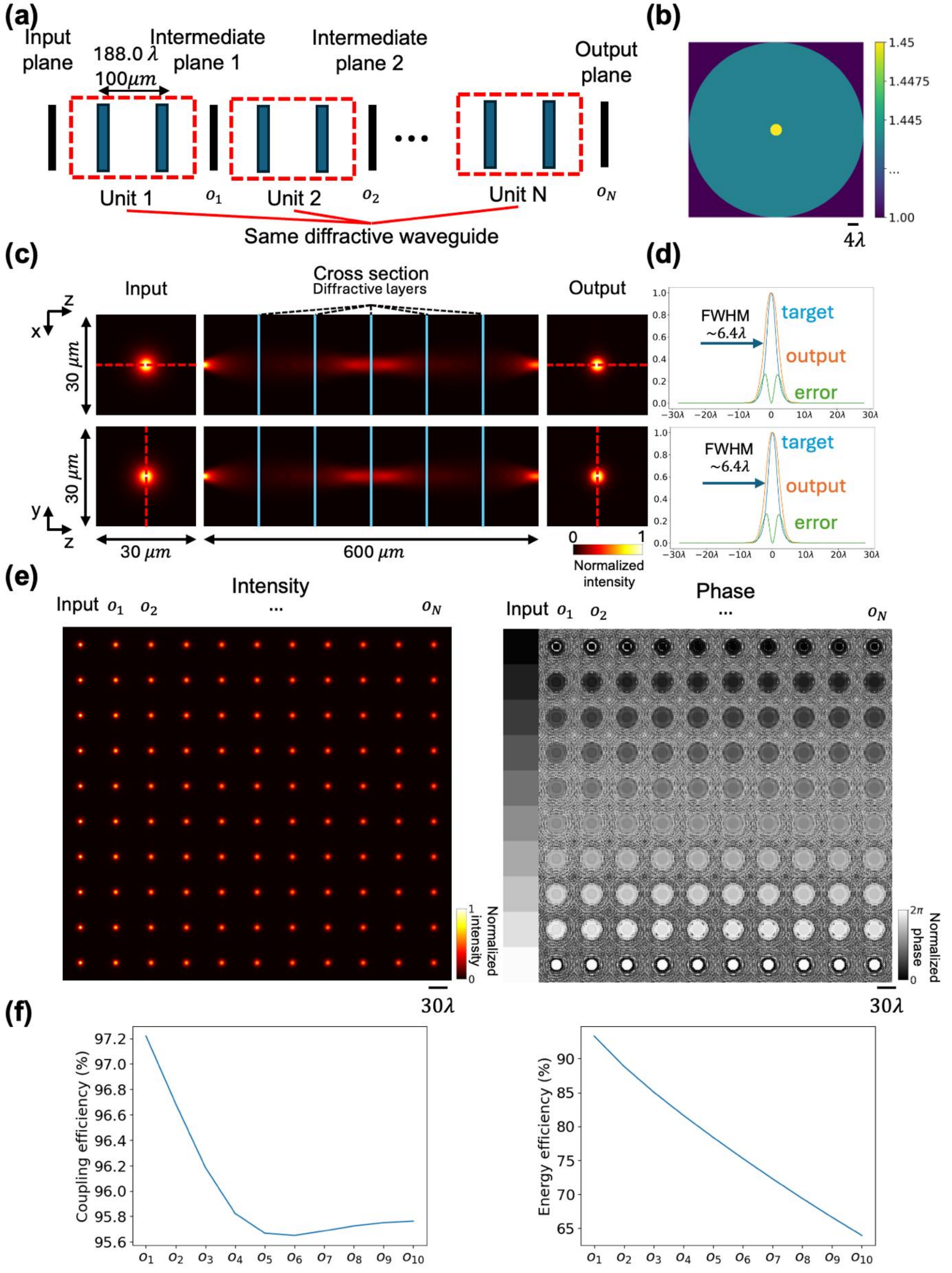

Supplementary Fig. S7. Testing results of a cascadable single-mode diffractive waveguide for visible optical mode transmission at $532\ nm$. (a) Schematic of a compact cascaded diffractive waveguide for

visible mode transmission at $532\,nm$ with N identical units. (b) Refractive index profile of the designed dielectric waveguide. (c) Cross sectional profile of the optical mode within a cascaded diffractive waveguide. (d) Output optical field profiles of the dielectric waveguide (target), the diffractive waveguide (output) and the error in between. (e) Intensity and phase profiles of optical fields at the input, intermediate and output planes. (f) Coupling efficiency and energy efficiency of the transmitted optical modes at the intermediate planes and the output plane.

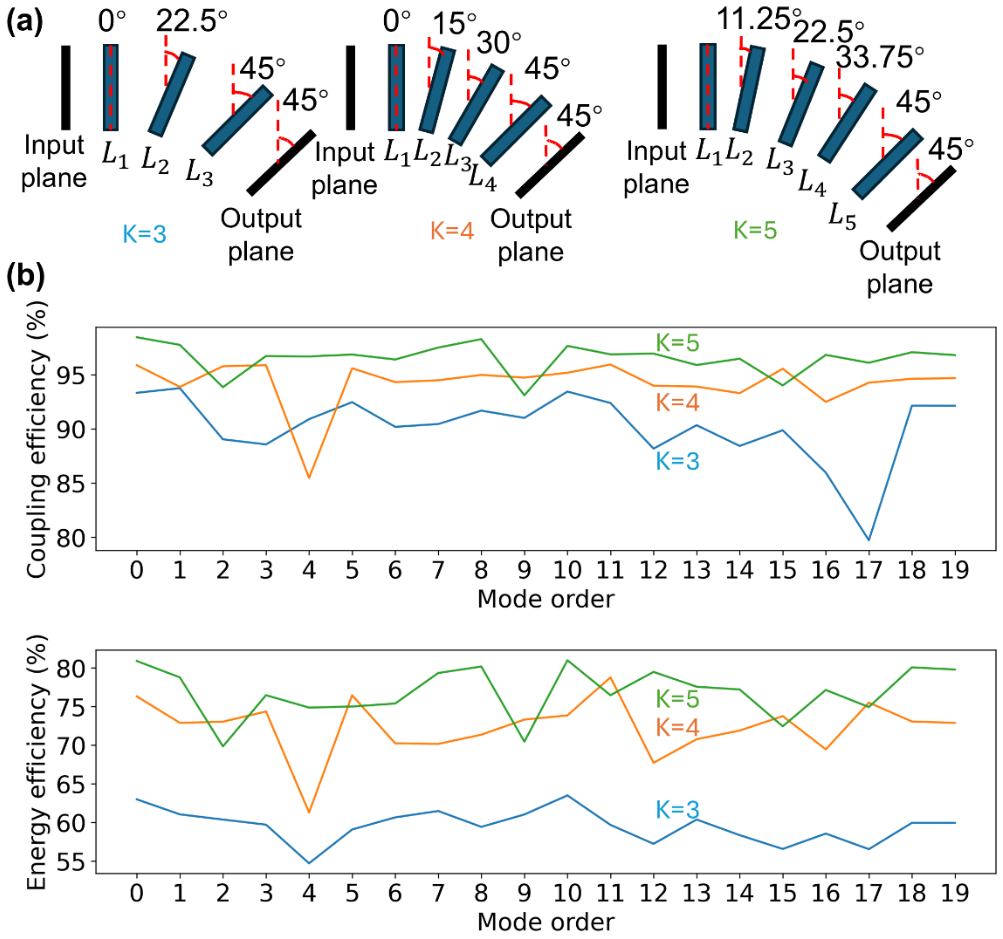

Supplementary Fig. S8. Testing results of different bent diffractive waveguide designs with the same total bending angle. (a) Schematics of bent diffractive waveguides with the same bending angle with different number of layers (*K*=3, 4, and 5). (b) Coupling efficiency and energy efficiency of bent diffractive waveguide designs with different number of diffractive layers.

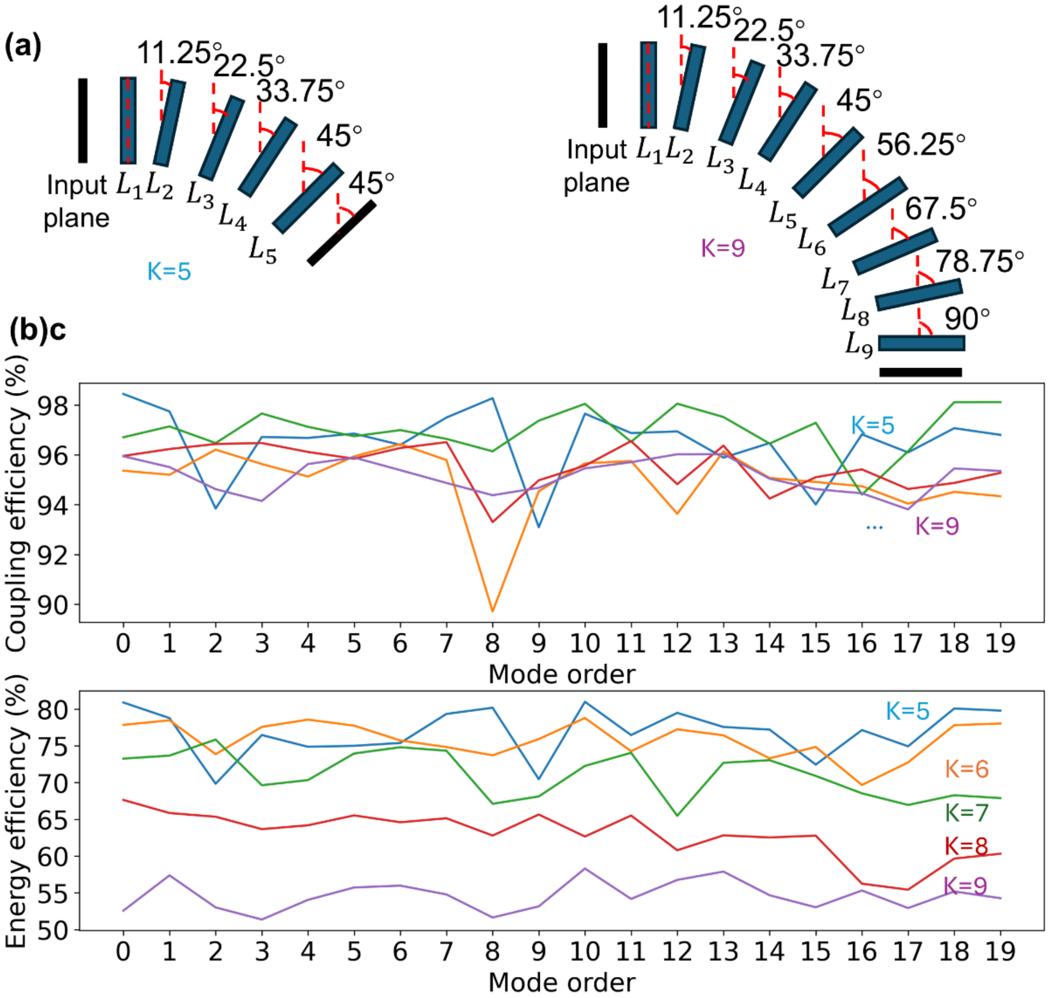

Supplementary Fig. S9. Testing results of different bent diffractive waveguide designs with the same bending angle between successive layers. (a) Schematic structure of different bent diffractive waveguide designs with the same bending angle between successive diffractive layers resulting in different total bending angles between the input and output planes. (b) Coupling efficiency and energy efficiency of bent diffractive waveguide designs with different number of diffractive layers.

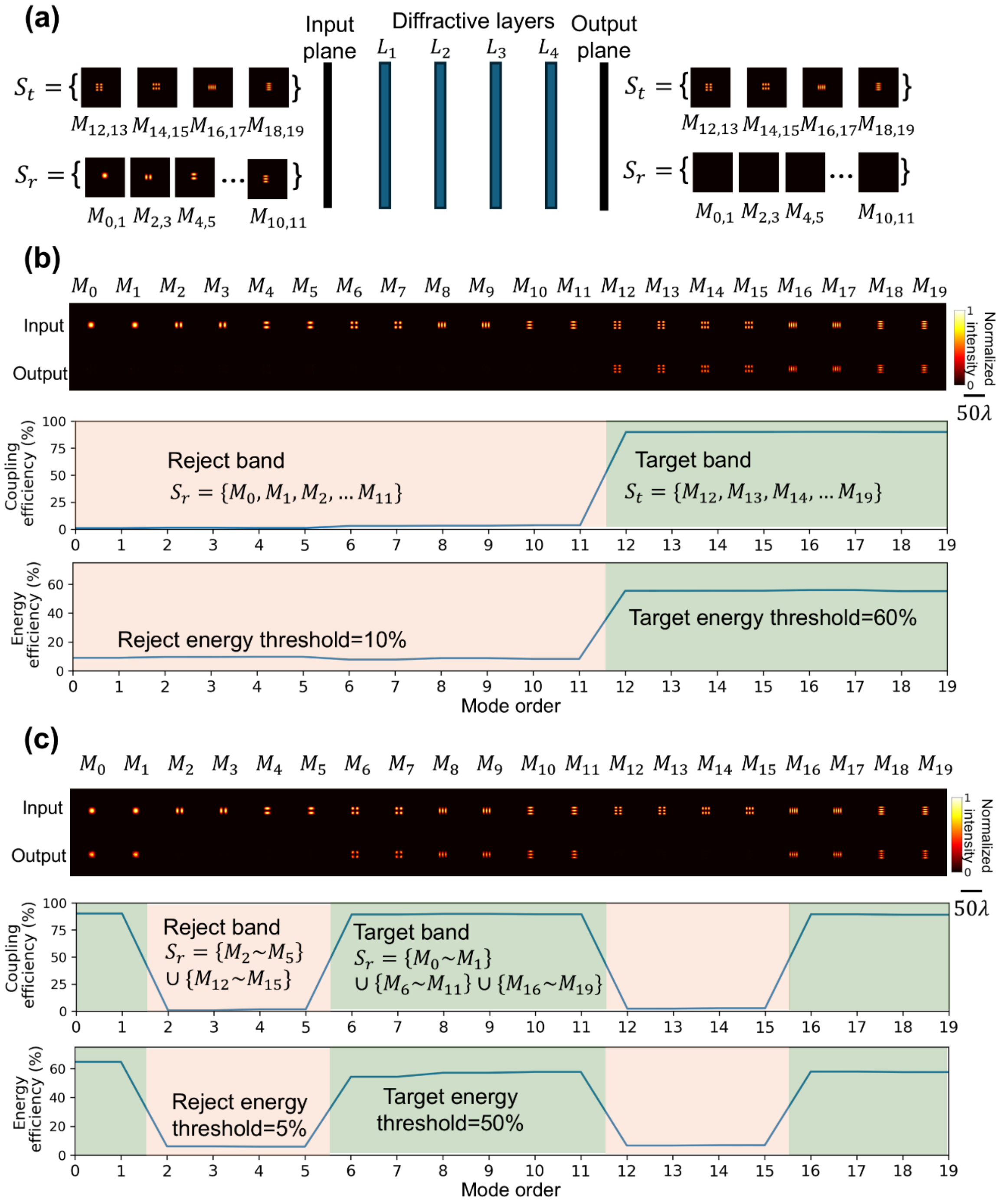

Supplementary Fig. S10. Testing results of a mode filtering diffractive waveguide. (a) Schematic of a mode filtering diffractive waveguide designed to pass the guided modes in the target set S_t while filtering out the modes in the rejection set S_r. Output fields, coupling efficiency and energy efficiency of (b) a high pass mode filtering diffractive waveguide, and (c) a bandpass mode filtering diffractive waveguide. Each row of output images is separately normalized.

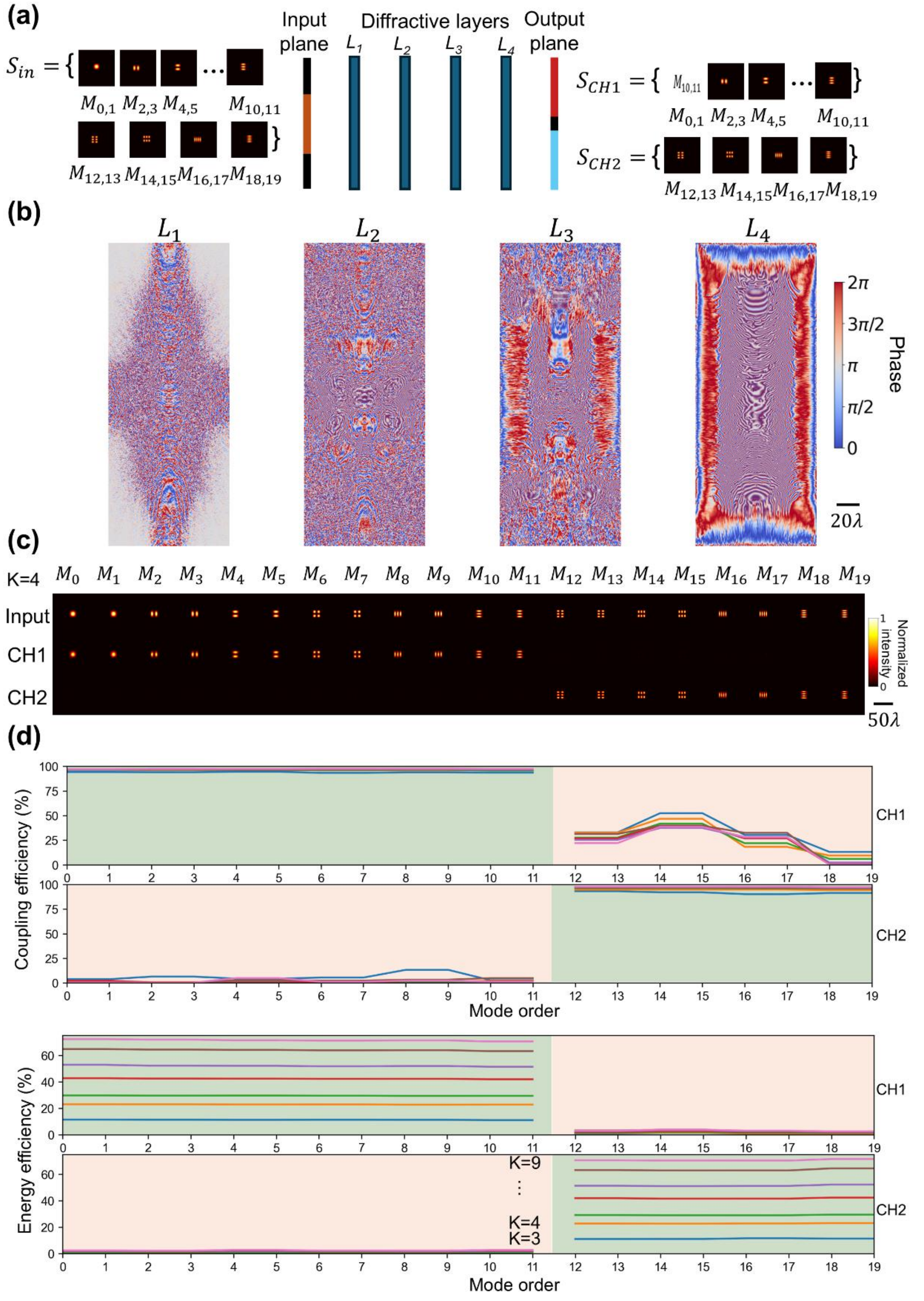

Supplementary Fig. S11. Testing results of a mode splitting diffractive waveguide. (a) Schematic of a mode splitting diffractive waveguide, designed to perform mode splitting from the input FOV to separate regions in the output FOV, depending on the order of the spatial modes. (b) Phase modulation patterns in

the converged diffractive layers of the mode splitting diffractive waveguide. (c) Blind testing results of the intensity profiles of the input fields and the output fields at the two output channels of the mode splitting diffractive waveguide. Each row of output images is separately normalized. (d) Coupling efficiency and energy efficiency of the two channels for the transmitted modes $\{M_0, M_1, \dots, M_{19}\}$ at the output plane. Mode splitting diffractive waveguides with different number of layers ($K = 3,4,..,9$) were trained and evaluated.

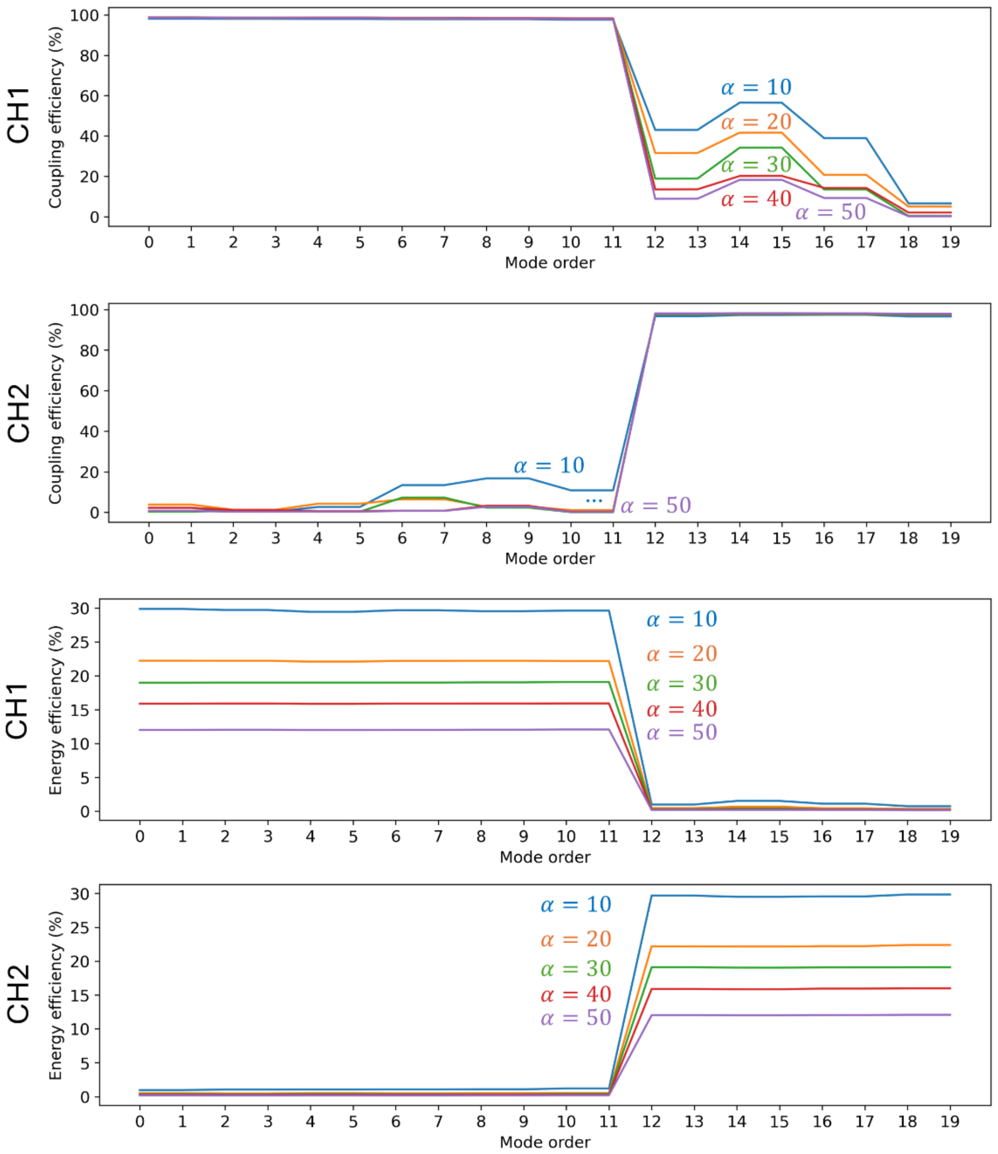

Supplementary Fig. S12. Coupling efficiency and energy efficiency of the two output channels of the diffractive mode splitting waveguide designs with different hyperparameters (α).

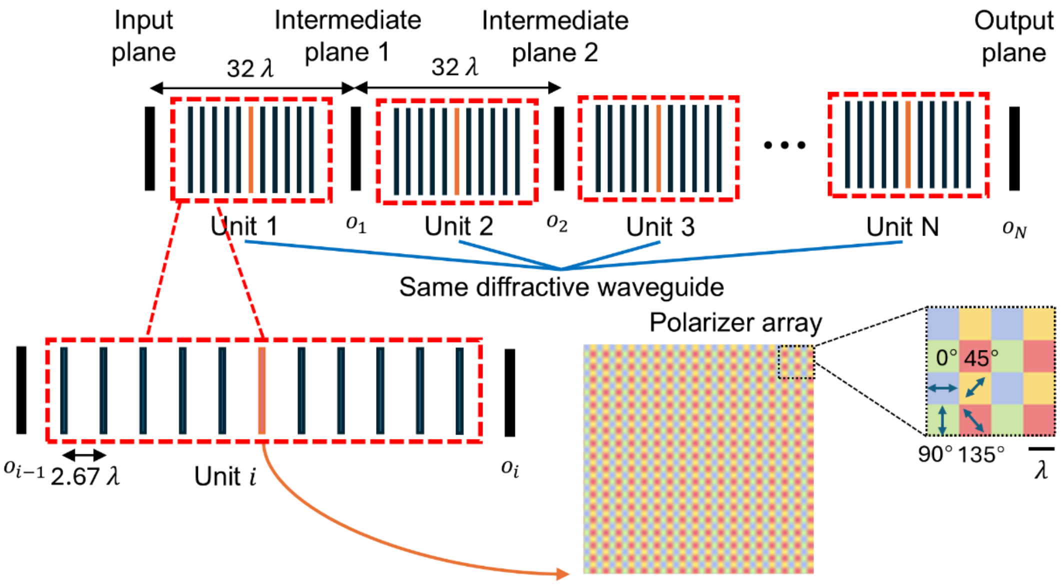

Supplementary Fig. S13. Schematic of a cascaded mode-specific polarization maintaining diffractive waveguide with *N* identical polarization maintaining diffractive units that are cascaded to each other.

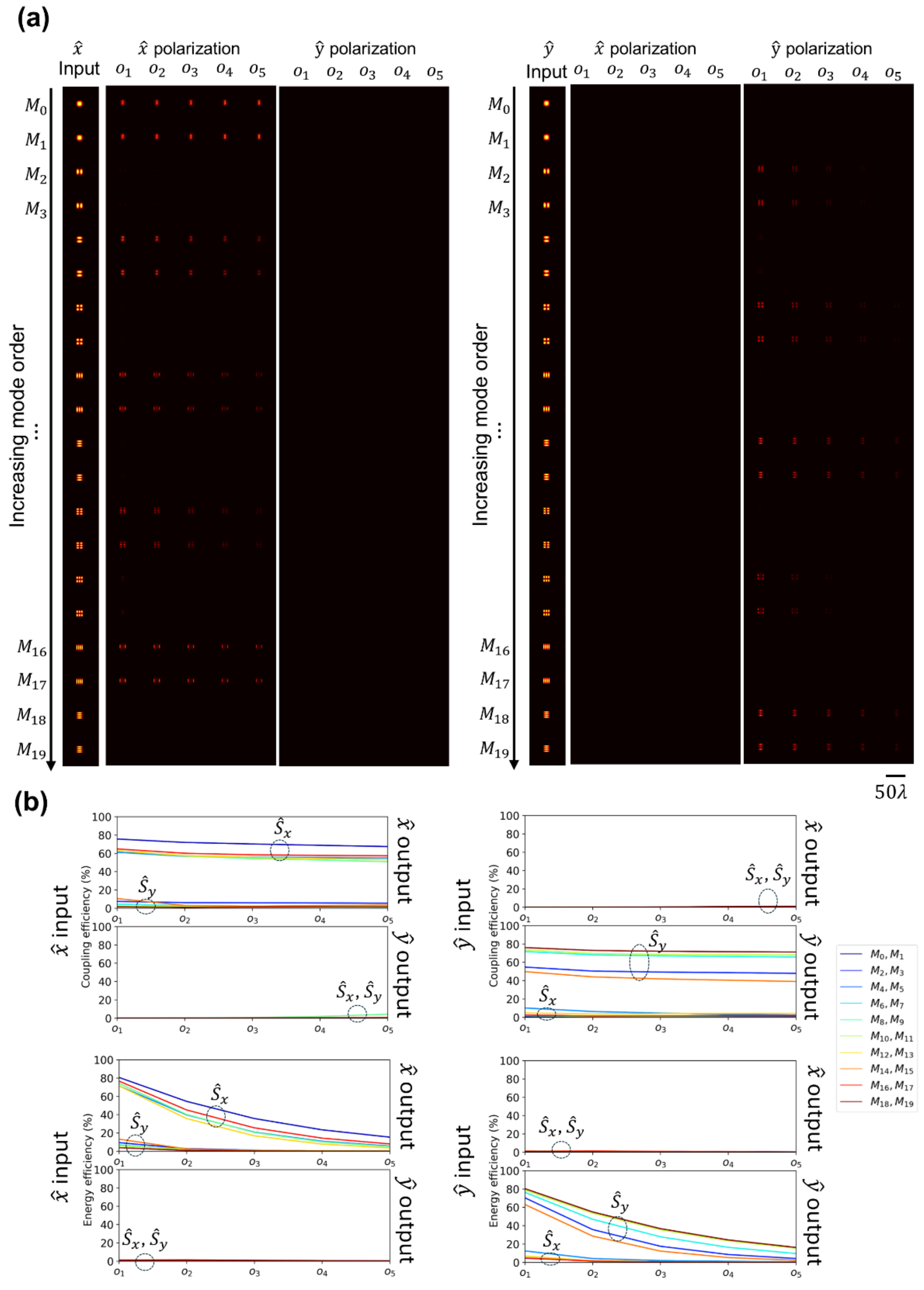

Supplementary Fig. S14. Testing results of a cascaded mode-specific polarization maintaining diffractive waveguide. (a) Intensities at the input, intermediate and output planes of all combinations of input and output polarization directions. (b) Coupling efficiency and energy efficiency of optical fields at the input, intermediate and output planes for all combinations of input and output polarization directions.

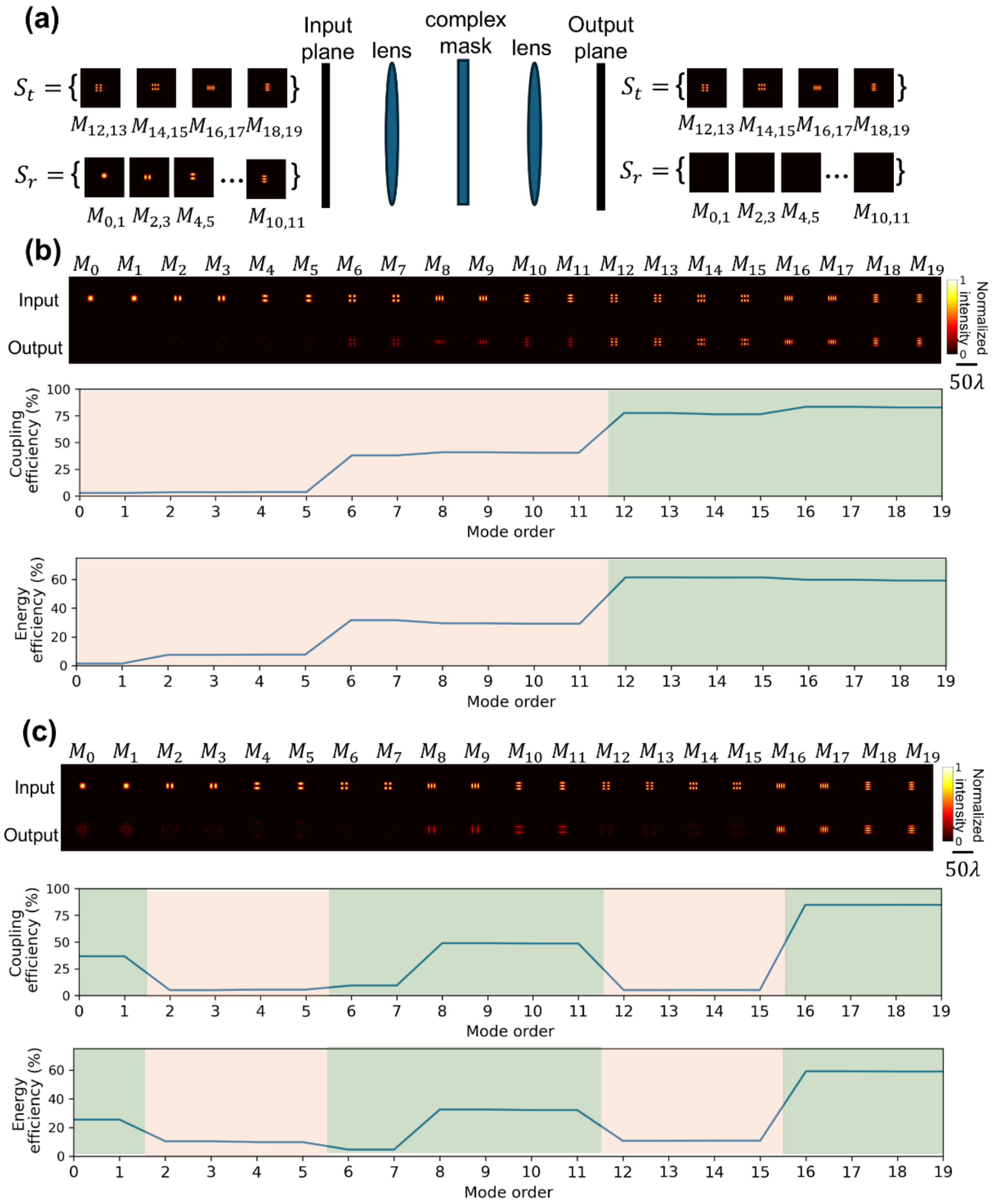

Supplementary Fig. S15. Testing results of a learnable 4-f system-based mode filtering design. (a) Schematic diagram for implementing the same functionalities proposed in Supplementary Fig. S10 of the main text using a learnable 4-f system. Output fields, coupling efficiency and energy efficiency of (b) a high pass mode filtering system using a learnable 4-f system, and (c) a bandpass mode filtering system using a learnable 4-f system.

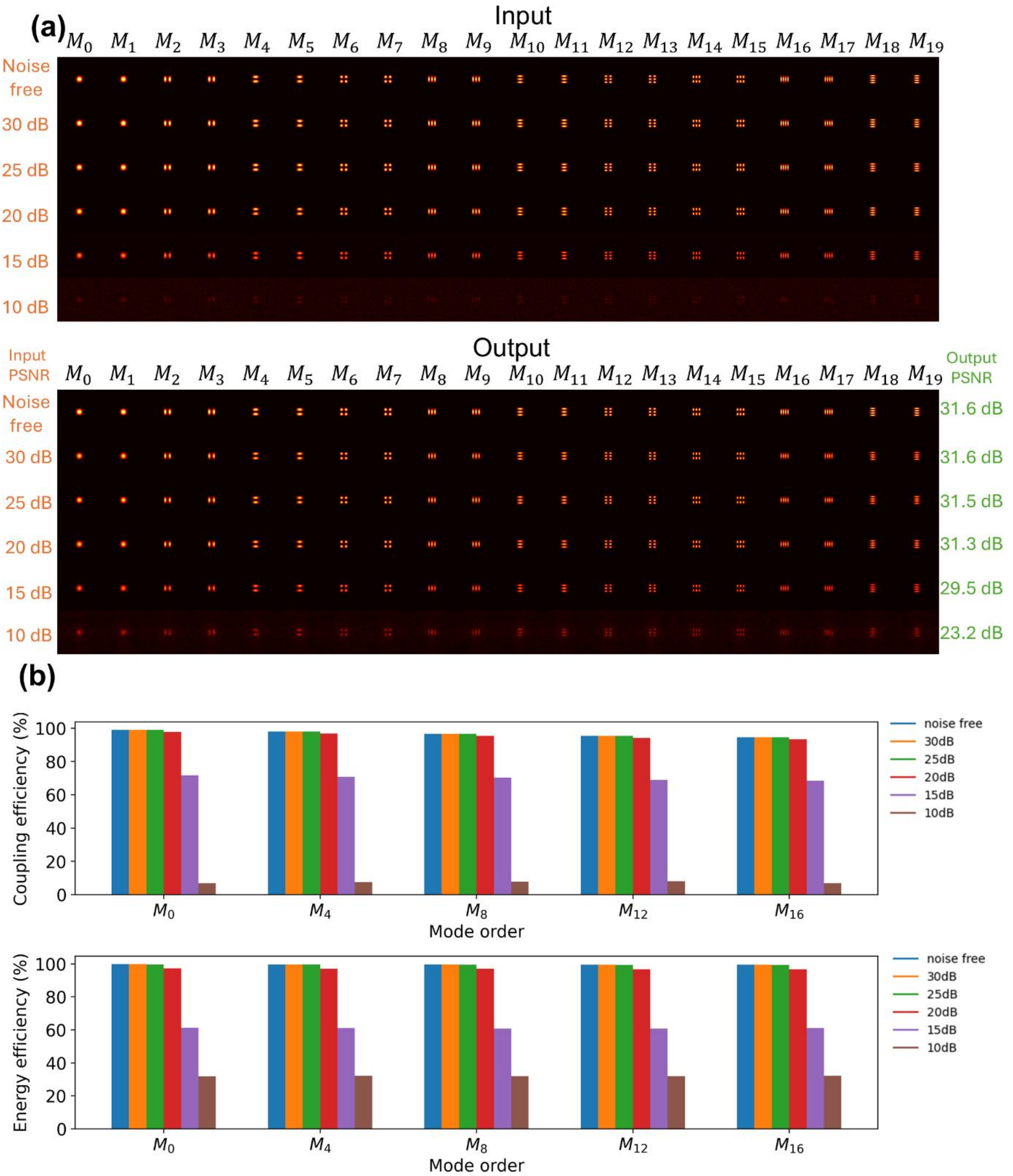

Supplementary Fig. S16. Testing results of diffractive waveguides against input noise. (a) Input and output profiles of various spatial modes with different PSNR levels. (b) Coupling and energy efficiency of several transmitted spatial modes at different levels of input noise.
